# Nasal carriage of *Staphylococcus aureus* and hygiene practices among food handlers in Skopje, North Macedonia

**DOI:** 10.2478/aiht-2026-77-4035

**Published:** 2026-06-30

**Authors:** Jansun Bukovetz, Jovan Todorovski, Gyltene Zendeli, Dragan Mijakoski, Mihail Kočubovski, Vesna Kotevska, Igor Spiroski

**Affiliations:** Institute of Public Health of the Republic of North Macedonia, Skopje, North Macedonia; Centre of Public Health Bitola, Bitola, North Macedonia; Institute of Occupational Health of the Republic of North Macedonia, Skopje, North Macedonia; Ss. Cyril and Methodius University in Skopje, Faculty of Medicine, Skopje, North Macedonia; Ss. Cyril and Methodius University in Skopje, Faculty of Medicine, Institute of Microbiology and Parasitology, Skopje, North Macedonia

**Keywords:** food safety, MRSA, MSSA, MRSA, MSSA, sigurnost hrane

## Abstract

Nasal carriage plays a key role in *Staphylococcus aureus* transmission, particularly among individuals working in the food sector. The aim of this cross-sectional study was to investigate the relationship between personal hygiene and nasal carriage of *S. aureus* among workers in food production, distribution, and trade. It was conducted in Skopje from November 2021 to March 2022 and included 289 workers undergoing mandatory health and hygiene examinations. Data were collected through a structured questionnaire and microbiological testing of nasal swabs. The overall prevalence of *S. aureus* nasal carriage was 12.5 % (95 % CI: 8.7–16.3 %). Methicillin-susceptible *S. aureus* (MSSA) prevailed in 11.8 % (95 % CI: 8.1–15.5 %) and the methicillin-resistant variety (MRSA) in only 0.7 % (95 % CI: 0–1.7 %). We found no significant associations between nasal carriage and demographic or hygiene variables (assessed using the chi-squared test) but did observe higher carriage rates among men (15.7 %), cooks (28.6 %), and individuals with untidy nails (17 %). However, our findings should be interpreted with caution, and future studies should address the limitations of the present study. Targeted training, routine screening, and consistent adherence to good hygiene practices remain important for minimising colonisation and reducing the risk of *S. aureus* transmission.

## Abstract (hr)

Prisutnost bakterije *Staphylococcus aureus* u nosu ima ključnu ulogu u njezinoj transmisiji, osobito među osobama zaposlenima u prehrambenom sektoru. Cilj ovog presječnog istraživanja bio je ispitati povezanost osobnih higijenskih navika i nosnog kliconoštva bakterije *S. aureus* među radnicima u proizvodnji, distribuciji i prometu hrane. Istraživanje je provedeno u Skoplju od studenoga 2021. do ožujka 2022. te je uključilo 289 radnika podvrgnutih obveznim zdravstvenim i higijenskim pregledima. Podaci su prikupljeni strukturiranim upitnikom i mikrobiološkim testiranjem obrisaka nosa. Ukupna prevalencija nosnog kliconoštva bakterije *S. aureus* iznosila je 12,5 % (95 %-tni interval pouzdanosti: 8,7–16,3 %). Meticilin-osjetljivi *S. aureus* (MSSA) zabilježen je u 11,8 % ispitanika (95 %-tni interval pouzdanosti: 8,1–15,5 %), a meticilin-rezistentni *S. aureus* (MRSA) u svega 0,7 % ispitanika (95 %-tni interval pouzdanosti: 0–1,7 %). Nije utvrđena statistički značajna povezanost između nosnog kliconoštva i demografskih odnosno higijenskih varijabli procijenjenih hi-kvadrat testom, no više stope kliconoštva zabilježene su među muškarcima (15,7 %), kuharima (28,6 %) i osobama s neurednim noktima (17 %). Iako nisu utvrđene statistički značajne povezanosti, uočene razlike upućuju na važnost kontinuiranog praćenja higijenskih navika i jačanja preventivnih mjera u prehrambenom sektoru.

Globally, nearly 600 million people fall ill each year due to contaminated food, resulting in 420,000 deaths (1). One of the most common foodborne pathogens is *Staphylococcus aureus*, which produces 21 known enterotoxins and exhibits increasing resistance to antibiotics (2, 3). In about 30 % of the human population who are persistent carriers, nostrils act as the primary reservoir, and hands as the main vector for transmission, although airborne dissemination may also contribute to disease outbreaks (4).

Food contamination with *S. aureus* can lead to staphylococcal food poisoning, especially in vulnerable groups, characterised by nausea, vomiting, and abdominal cramps, which typically develop within 30 min to 8 h and resolve within 24–48 h (5, 6). Most often, food contamination occurs in dairy products, cereals, processed meats, and ready-to-eat meals (7, 8), which points to food handlers, nasal carriers in particular, as the most likely source (9, 10).

However, information about nasal carriage of *S. aureus* among workers involved in food production, distribution, and trade in the Republic of North Macedonia is scarce, even though they undergo monitoring every six months. The aim of our study was therefore to address this gap and to investigate the relationship between nasal carriage of *S. aureus* and personal hygiene practices in this population.

## PARTICIPANTS AND METHODS

This cross-sectional study was conducted at the Institute of Public Health in Skopje, North Macedonia, between November 2021 and March 2022 as part of mandatory health and hygiene examinations of workers employed in food production, distribution, and trade. It included 289 participants (140 men and 149 women) who voluntarily agreed to participate, completed the questionnaire, and gave written informed consent prior to inclusion in the study.

Data collection included microbiological testing and a structured questionnaire. A single nasal swab was obtained from each participant and immediately inoculated onto blood agar plates. Plates were incubated at 35–37 °C for 24 h. Presumptive staphylococcal colonies were identified based on colony morphology and Gram staining, which demonstrated Gram-positive cocci arranged in grape-like clusters. Catalase testing was performed to confirm the genus *Staphylococcus*, while *S. aureus* was differentiated from coagulase-negative staphylococci (CoNS) using the DNase test.

Methicillin resistance was determined phenotypically using the cefoxitin (30 µg) disc diffusion method on Mueller-Hinton agar. Inhibition zone diameters were measured and interpreted according to the European Committee on Antimicrobial Susceptibility Testing (EUCAST) guidelines (11). Isolates with inhibition zones <22 mm were classified as methicillin-resistant *S. aureus* (MRSA), whereas isolates with inhibition zones ≥22 mm were classified as methicillin-sensitive *S. aureus* (MSSA). Phenotypic testing is the standard used in routine public health surveillance and screening programmes.

The questionnaire included questions on general demographic characteristics, health status, social activities, personal hygiene practices, work habits, and home hygiene. The sections concerning general demographic characteristics and health status were completed with the assistance of a physician, whereas the sections related to social activities, hygiene practices, work habits, and home hygiene were self-completed by the participants at their workplaces.

### Statistical analysis

Statistical analyses were performed using SPSS version 16.0 (SPSS Inc., Chicago, IL, USA). Associations between nasal carriage of *S. aureus* and categorical variables were assessed using the chi-squared test. Prevalence estimates were calculated with 95 % confidence intervals (CI). A p-value <0.05 was considered statistically significant.

## RESULTS

Among the 289 food handlers, 36 (12.5 %, 95 % CI: 8.7–16.3 %) were nasal carriers of *S. aureus*; 34 (11.8 %, 95 % CI: 8.1–15.5 %) of MSSA, and two (0.7 %, 95 % CI: 0–1.7 %) of MRSA (Table 1).

**Table 1:** Prevalence of MSSA and MRSA nasal carriage among food handlers in Skopje (with 95 % CI)

**Category**	**N**	**Prevalence (%)**	**95 % CI**
MSSA and MRSA	36	12.5	8.7–16.3
MSSA	34	11.8	8.1–15.5
MRSA	2	0.7	0–1.7

MRSA – methicillin-resistant *S. aureus*; MSSA – methicillin-sensitive *S. aureus*

We found no statistically significant associations between *S. aureus* nasal carriage and any of the investigated parameters, including gender, job, body mass index, tooth brushing frequency, facial hair presence, nail hygiene, nose picking, nose rinsing, and hand washing (Table 2).

**Table 2:** Nasal *S. aureus* carriage association with gender, job, body mass index, tooth brushing frequency, facial hair presence, nail hygiene, nose picking, nose rinsing, and hand washing

**Variable**	**Nasal carriers**		**Non-carriers**		**Total**	**Chi-square**	**df**	**p**
	**n**	**%**	**n**	**%**	**n**			
**Gender**								
Male	22	15.7	118	46.6	140	2.642	1	0.104
Female	14	9.4	135	53.4	149			
Total	36	12.5	253	100	289			
**Job**								
Cook, baker, assistant cook	2	28.6	5	71.4	7	5.676	9	0.772
Distributor	1	16.7	5	83.3	16			
Quality control	0	0	19	100	19			
Storekeeper	9	15.3	50	84.7	59			
Packager	1	11.1	8	88.9	9			
Salesman	0	0	1	100	1			
Worker in production	19	13.1	126	86.9	145			
Caterer	2	10	18	90	20			
Cleaner	1	12.5	7	87.5	8			
Worker	1	6.7	14	93.3	15			
**Body mass index**								
Underweight	1	25	3	75	4	8.736	5	0.120
Normal weight	9	8.3	99	91.7	108			
Overweight	12	10.5	102	89.5	114			
Obesity class I	10	21.7	36	78.3	46			
Obesity class II	4	25	12	75	16			
Obesity class III	0	0	1	100	1			
**Daily tooth brushing frequency**								
0	8	17.8	37	82.2	45	6.546	4	0.162
1	12	16.7	60	83.3	72			
2	11	8	126	92	137			
3	3	10	25	89.3	28			
≥4	2	28.6	5	71.4	7			
**Facial hair**								
Beard	4	9.5	38	90.5	42	0.388	1	0.534
No beard	32	13	215	87	247			
Moustache	4	11.1	32	88.9	36	0.068	1	0.794
No moustache	32	12.6	221	87.4	253			
**Nail hygiene**								
Tidy	28	11.6	214	88.4	242	1.294	1	0.255
Untidy	8	17	39	83	47			
**Nose picking frequency**								
Often	3	17.6	14	82.4	17	2.250	3	0.522
Rarely	9	17.6	42	82.4	51			
Very rarely	9	10.2	79	89.8	88			
Not at all	15	11.3	118	88.7	133			
**Nasal rinsing before work**								
Yes	13	12.5	91	87.5	104	2.061	2	0.357
No	19	14.7	110	85.3	129			
Sometimes	4	7.1	52	92.9	56			
**Nasal rinsing after work**								
Yes	11	12.8	75	87.2	86	0.194	2	0.907
No	19	12.9	128	87.1	147			
Sometimes	6	10.7	50	89.3	56			
**Hand washing after using restroom**								
Yes	34	12	249	88	283	2.449	1	0.118
Sometimes	2	33.3	4	66.7	6			
**Hand washing with soap after nose picking**								
Yes	26	12.6	180	87.4	206	1.325	2	0.515
No	5	9.1	50	90.9	55			
Sometimes	5	17.9	23	82.1	28			

However, higher nasal carriage prevalence was observed among men, cooks, bakers, and assistant cooks, participants with untidy nails, those who reported never picking their nose, those not rinsing their nose before or after work, and those who only sometimes washed their hands after using the toilet or after nose picking.

## DISCUSSION

Overall, nasal *S. aureus* carriage was consistently present among food handlers, with slight variations across gender, job category, body mass index, and hygiene habits, but the differences were not statistically significant. The overall prevalence of 12.5 % falls within the range reported across countries and occupational groups. Two studies from Turkey (14, 15) reported MSSA prevalence rates of 3.37 % and 15.25 % and MRSA rates of 5.3 % and 2.6 %, respectively. In Portugal (12), MSSA prevalence rate was 19.8 %, while in Greece (16) MSSA and MRSA rates were 13.8 % and 0.6 %, respectively. Studies from Ethiopia (17) documented an 11.3 % prevalence of *S. aureus*. In the United States of America (18), MSSA and MRSA were reported at the rates of 23.3 % and 3.6 %, respectively, and in Hong Kong (19), the overall prevalence of *S. aureus* was reported at 22.9 %.

Although Beyene et al. (17) reported a significant association between cooks and *S. aureus* carriage (p=0.00336), our results are not in line with this finding and suggest that occupational exposure may not be the sole determinant of nasal colonisation in this setting.

Regarding gender, our study found no statistically significant difference between men and women, which is consistent with the findings reported by Beyene et al. (17). However, a slightly higher prevalence was observed among men, in line with previous population-based studies reporting male sex as a risk factor for nasal *S. aureus* carriage (20).

Furthermore, our findings do not support reports of obesity as a risk factor for *S. aureus* nasal carriage, potentially due to impaired immune function, altered microbiome, and chronic inflammation (21).

Research on the specific contribution of facial hair among food handlers is limited, but facial hair management may influence bacterial colonisation. Our findings are consistent with Wakeam et al. (22), who found no association between facial hair and nasal carriage of *S. aureus* among health workers. Similarly, a Turkish study reported no significant differences in carriage between men with or without a moustache (23).

According to good hygiene practice standards, food handlers have to maintain good hand hygiene (24), which is critical in reducing the risk of *S. aureus* colonisation and transmission, hand washing in particular (25). While our study found no significant association between hand washing and nasal carriage, higher carriage rates were observed among participants who only sometimes washed their hands after nose picking. In addition, nail condition also showed no significant association, although participants with untidy nails had slightly higher carriage rates.

Nose picking has been reported as a risk factor for nasal carriage, with Wertheim et al. (26) reporting higher colonisation among frequent pickers. Although no statistically significant association was observed in our study, the highest carriage rate was identified among participants who reported picking their nose often and rarely.

Oral hygiene may also play a role in nasal *S. aureus* colonisation, as the oral cavity is a common bacterial reservoir (27, 28). However, toothbrushing frequency was not significantly associated with nasal carriage in our sample.

Nasal rinsing, particularly with water or saline solutions, has been shown to reduce *S. aureus* carriage by physically removing bacteria, disrupting biofilms, and inhibiting bacterial growth (29, 30). Although not statistically significant in our study, slightly lower carriage rates were observed among participants who reported rinsing their noses.

### Study limitations

Our study has several limitations. First, the presence of *S. aureus* in the nasal cavity does not necessarily indicate enterotoxin production, and we did not determine classical enterotoxin genes (*sea, seb, sec, sed, see*). Second, laboratory analyses relied on culture-based and phenotypic methods for identifying MSSA and MRSA, while molecular confirmation of resistance mechanisms (e.g. *mecA* PCR) was not performed, as this is not the scope of routine health and hygiene surveillance programs. Although PCR detection of the *mecA* gene is considered the molecular gold standard, phenotypic detection using the cefoxitin disc diffusion method has shown high agreement with PCR results and remains a reliable and widely accepted method for routine MRSA screening (31). Third, self-reporting necessarily entails the risk of bias. Fourth, the study was conducted in Skopje over five months and may not capture seasonal variations or reflect all regions of the country. Data on specific work practices which may influence nasal colonisation risk, including contact with raw products or shift duration, were not detailed in the questionnaire. Finally, our study focused on nasal carriage without linking it to clinical outcomes or food contamination events. These considerations provide context for interpreting the findings and may guide future research in the field.

## CONCLUSION

Our study showed a 12.5 % nasal carriage prevalence of *S. aureus* among food handlers and no significant associations with gender, BMI, facial hair, nail condition, or specific hygiene practices, although slightly higher carriage rates were observed among men, cooks, and individuals with untidy nails. However, our findings should be interpreted with caution, and future studies should address the limitations of the present study. Targeted training, routine screening, and consistent adherence to good hygiene practices remain important for minimising colonisation and reducing the risk of *S. aureus* transmission.
